# Blastic plasmacytoid dendritic cell neoplasm presenting with a cutaneous tumor alone as the first symptom of onset: A case report and review of literature

**DOI:** 10.3892/ol.2014.2759

**Published:** 2014-12-03

**Authors:** GUOHUA YU, WEI WANG, YEKUN HAN, JING LIU, XUBO PAN, GUIMEI QU

**Affiliations:** 1 Department of Pathology, Affiliated Yantai Yuhuangding Hospital, Medical College of Qingdao University, Yantai, Shandong, P.R. China; 2 Department of Pharmacology, Affiliated Hospital, Binzhou Medical College, Binzhou, Shandong, P.R. China

**Keywords:** blastic plasmacytoid dendritic cell neoplasm, cutaneous involvement, differential diagnosis

## Abstract

Blastic plasmacytoid dendritic cell neoplasm (BPDCN), formerly named cluster of differentiation (CD)4^+^/CD56^+^ haematodermic neoplasm or blastic natural killer cell lymphoma, is a rare and highly aggressive haematopoietic malignancy. BPDCN typically occurs in the elderly, with a marked predilection for cutaneous involvement. The present study describes a case of BPDCN occurring in a 79-year-old male. The patient presented with skin lesions alone, with no evidence of extracutaneous involvement during the course of the disease. BPDCN was diagnosed based on histological and immunohistochemical observations and the patient was subsequently treated with local radiotherapy alone. However, rapid disease progression occurred and the patient succumbed five months after being diagnosed. The current result therefore demonstrated that BPDCN is highly aggressive even without systemic dissemination, and that radiotherapy appears to be ineffective in treating this tumor. The present study emphasizes the importance of pathologists and dermatologists being aware of this uncommon disease in order to avoid misdiagnosis.

## Introduction

Blastic plasmacytoid dendritic cell neoplasm (BPDCN) is an extremely rare haematological malignancy that was recently considered as a distinct disease entity originating from the precursors of plasmacytoid dendritic cells (PDCs) (1). Patients with BPDCN are usually in their 50s and 60s (2,3), but children can also be affected (4–6). Skin lesions typically appear as the first clinical manifestation, with frequent evolution towards overt leukemia (7). Histologically, BPDCN presents as a diffuse monomorphic infiltrate of medium-sized lymphoid cells in the dermis and the subcutis, with an uninvolved zone of collagen band (Grenz zone) separating the infiltrate from the epidermis (8,9). The characteristic immunophenotypic profile of BPDCN is positivity for cluster of differentiation (CD)56, CD4 and CD123, with a usually negative result for other T-cell, B-cell and myeloid cell markers (2,3). At present there is no curative treatment available for BPDCN and its prognosis is usually poor (10,11). The present study describes a case of BPDCN in a 79-year-old male and reviews the differential diagnoses, treatment and prognosis of BPDCN to further highlight this rare entity.

## Case report

### Case presentation

**Figure 1:**
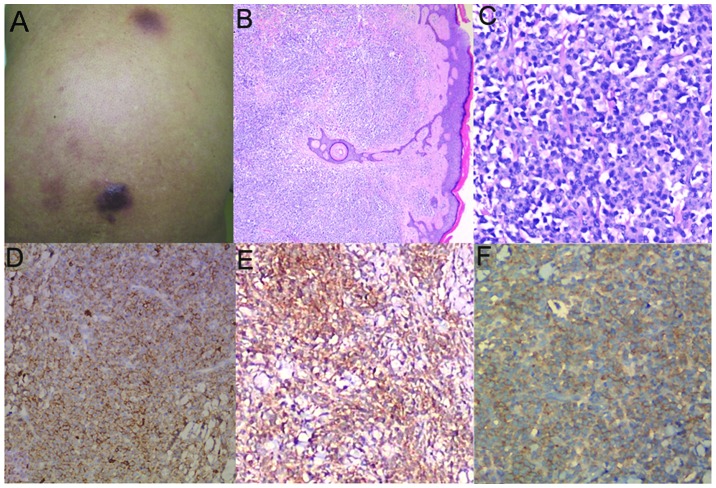
Macroscopic, microscopic and immunophenotying findings. (A) Gross image showing a reddish papule and a violaceous nodule, each 3 cm in diameter, on the upper and lower mid back, respectively. Hematoxylin and eosin (H&E) staining showing (B) a normal epidermis and a diffuse cellular infiltration involving the dermis and subcutis, with a homogeneous and acellular band between tumor compositions and epidermis (magnification, ×40); and (C) medium-sized tumor cells, with scarce cytoplasm, finely dispersed chromatin and inconspicuous nucleoli (magnification, ×200). Immunophenotypic examination revealed that the tumor cells were (D) cluster of differentiation (CD)4-positive, (E) CD56-positive, and (F) CD123-positive (Envision, ×200; Dako, Glostrup, Denmark).

In May 2009, a 79-year-old male was admitted to the Department of Dermatology, Affiliated Yantai Yuhuangding Hospital, Medical College of Qingdao University (Yantai, China) subsequent to noting asymptomatic purple and brown plaques on his back that had been present for one month. The lesions grew progressively. Upon admission, a skin examination revealed a reddish papule of 3 cm in diameter and a violaceous nodule of 3 cm in diameter on the upper and lower mid back, respectively, without superficial ulceration (Fig. 1A). A skin biopsy was subsequently performed. Routine laboratory tests were within normal ranges. A bone marrow aspiration and biopsy were performed and revealed no evidence of tumor cells. Additionally, a chest computed tomography (CT) scan and abdominal ultrasonography did not reveal any other abnormalities.

### Pathological findings

The histological examination of the skin biopsy performed upon admission revealed that the reddish papule exhibited a dense and diffuse infiltrate of monomorphous medium-sized cells in the dermis and subcutis. An uninvolved zone of collagen band was observed in the upper dermis (Fig. 1B). The tumor cells exhibited round to ovoid nuclei, with fine chromatin, scanty cytoplasm and inconspicuous nucleoli (Fig. 1C).

### Immunohistochemical and in situ hybridization (ISH) findings

Immunohistochemistry showed that the tumor cells were positive for CD4, CD56, CD123 (Fig. 1D–F) and CD43, but negative for CD3, CD5, CD20, CD30, CD45RO, CD79α, CD68, anaplastic lymphoma kinase, paired box 5, myeloperoxidase, granzyme B, T-cell intracellular antigen-1 and terminal deoxynucleotidyl transferase (TdT). The investigations for Epstein-Barr virus (EBV) infection by ISH for EBV-encoded small RNAs yielded a negative result. The patient was treated with local radiotherapy alone (50 Gy total, 25 times over five weeks) and succumbed to multiple organ failure five months later, in October 2009.

Written informed consent was obtained from the patient for publication of this case study and any accompanying images.

## Discussion

BPDCN is an extremely uncommon disease entity that poses great diagnostic challenges for dermatologists and pathologists. Due to the rarity of this disease, the etiology, pathogenesis and natural history of BPDCN have not yet been fully elucidated.

BPDCN is typically a disease that occurs in the elderly, but it can also occur in children. Clinically, BPDCN is highly aggressive, with a median survival time of 12–14 months (10,11). The majority of patients with BPDCN experience disease rapid progression that spreads to the bone marrow, the blood and the organs or tissues (7). The patient in the present study presented with skin lesions as the first symptom of onset, with no evidence of extracutaneous involvement, however, rapid disease progression occurred prior to mortality. Therefore, it is necessary to rapidly determine the correct diagnosis and provide effective treatment.

BPDCN must be distinguished from the accumulation of plasmacytoid dendritic cells associated with acute myeloid leukemia. These two diseases possess great similarities regarding the clinical and histopathological features. Immunophenotypic analysis can be useful in this respect. BPDCN is usually positive for CD56, but negative for granzyme B, whereas in the cutaneous accumulation of plasmacytoid dendritic cells associated with acute myeloid leukemia, the results are the opposite (7). Another important differential diagnosis for BPDCN is cutaneous natural killer (NK)/T-cell lymphoma. In addition to the absence of EBV and cytotoxic molecules, a lack of patchy necrosis, vascular invasion and cellular pleomorphism are important in the histological morphology of BPDCN in order to distinguish the disease from NK/T-cell lymphoma (12). In recent years, further antigens associated with plasmacytoid dendritic cell neoplasms have been successively found, such as CD2-associated protein, T-cell leukemia 1, CD303/BDCA-2 and CD304/BDCA-4 (13,14). These markers could aid in differentiating BPDCN from myelocytic malignancies and T- or NK/T-cell lymphomas.

At present, there is no effective or uniform treatment approach for BPDCN. A radical treatment plan for acute leukemia was previously reported to be of some success in acquiring complete remission in certain BPDCN patients, however, these patients usually relapsed within several months (15). The patient in the present study was treated with radiotherapy and succumbed five months later. Pagano *et al* (16) suggested that systematic preventive intrathecal chemotherapy should be indicated in the treatment of BPDCN, as the central nervous system may be a persistent blast-cell sanctuary.

A few factors, including an age of <40 years (11), lesions restricted to the skin (17) and strong TdT expression in the tumor cells (18), have been reported to be associated with a more favorable prognosis. However, the underlying mechanisms by which these factors affect the prognosis remain unclear. The present patient did not show extracutaneous involvement, but experienced a rapidly progressive clinical course, with a short survival time. This suggested that lesions restricted to the skin are not adequately proven as an independent index for prognosis.

In summary, the present study reports a case of BPDCN presenting with cutaneous involvement alone in a patient who experienced rapid disease progress prior to mortality. Although numerous types of lymphoma or leukemia can primarily or secondarily involve the skin, BPDCN, despite its rarity, should also be considered in the differential diagnosis. Additionally, the present findings further highlight the ineffectiveness of conventional radiotherapy in treating this unique disease and also alerts clinicians to the requirement to establish treatment modalities.
